# Will expanding catastrophic coverage eligibility increase marketplace premium affordability in 2026?

**DOI:** 10.1093/haschl/qxaf202

**Published:** 2025-10-23

**Authors:** David M Anderson, Dylan Nagy, Coleman Drake

**Affiliations:** Department of Health Services, Policy and Management, University of South Carolina, Columbia, SC 27801, United States; Department of Health Policy and Management,University of Pittsburgh, Pittsburgh, PA 15261, United States; Department of Health Policy and Management,University of Pittsburgh, Pittsburgh, PA 15261, United States

**Keywords:** health insurance, Affordable Care Act, Obamacare, Health Insurance Marketplace, One Big Beautiful Bill Act, premium tax credit subsidy, uninsured, underinsurance

## Introduction

On September 4th, 2025, the Centers for Medicare and Medicaid Services (CMS) announced it would improve access to affordable health coverage by expanding access to catastrophic health insurance marketplace plans.^1,2^ Catastrophic plans have lower premiums, higher out-of-pocket costs, and are ineligible for marketplace premium tax credit subsidies (PTCs). Previously, enrollees under age 30 or those meeting limited hardship exemptions could enroll in catastrophic plans. Starting in 2026, any enrollee with an income above 250% of the federal poverty level (FPL), roughly half of the 25 million current marketplace enrollees,^3^ will receive an automatic hardship exemption and may purchase catastrophic plans.^1^

It is unclear whether expanding catastrophic plan eligibility will improve premium affordability, the minimum monthly cost of marketplace coverage, for two reasons.^4^ First, many marketplace enrollees can already purchase PTC-subsidized bronze plans that may have lower premiums. Second, expanded PTCs (ePTCs) from the Inflation Reduction Act—which decreased enrollees’ premiums and eliminated the original PTC income eligibility limit of 400% FPL—will expire on December 31st unless Congress votes to extend them.^5^

## Methods

We calculated differences between mean monthly premiums for the lowest premium catastrophic and subsidized bronze plans across counties in the 31 states that used healthcare.gov in 2025 and offered catastrophic plans, which were not always available. We calculated subsidized bronze premiums in scenarios where ePTCs are and are not extended. We did so for enrollees affected by new catastrophic eligibility rules—those ages 31 to 64 with incomes above 250% FPL.

We identified the lowest premium catastrophic and bronze plans offered in each county using the 2025 Qualified Health Plan Landscape File. We calculated these plans pre- and, where applicable, post-subsidy premiums using federal data on marketplace age rating, the 2025 FPL, 2025 expanded premium tax credit formulas, and 2026 non-expanded premium tax credit formulas. We weighted counties using 2025 enrollment above 250% FPL from CMS Open Enrollment Period Public Use File.^3^ See Supplementary material for details.

## Results

Of the 2155 counties in the 31 states that used healthcare.gov in 2025, catastrophic plans were available in 1420 counties in 21 states, which thereby constituted our sample. These counties contain 2.3 million marketplace enrollees with incomes above 250% FPL that will be newly eligible to purchase catastrophic plans.

**Figure 1.**
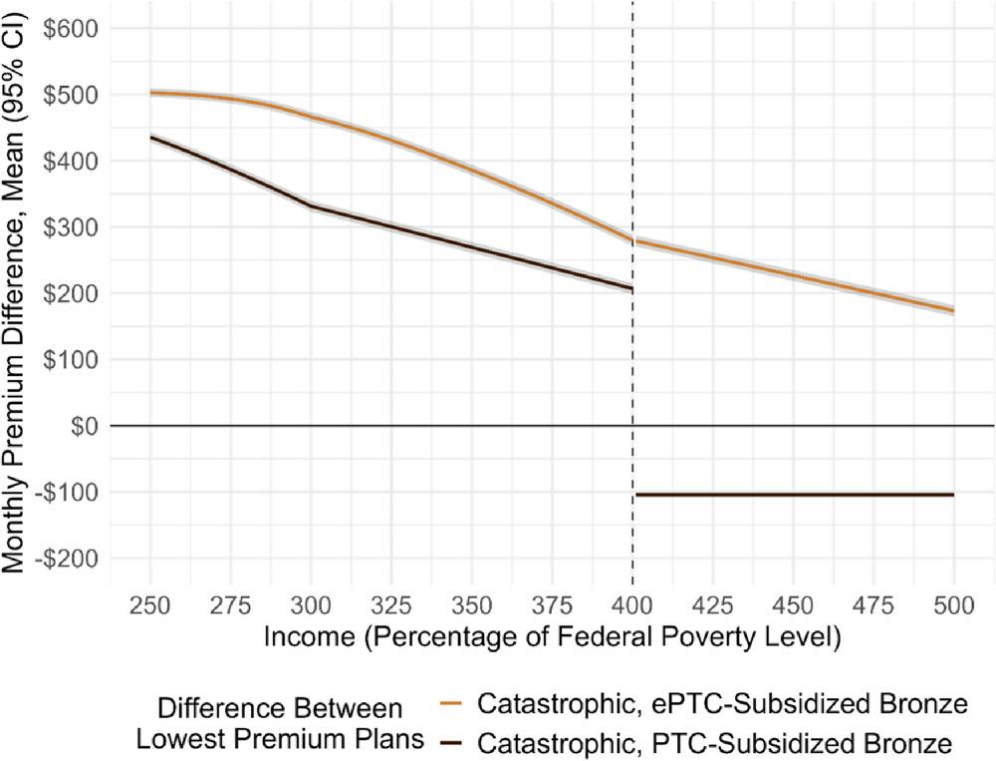
Differences between 2025 mean monthly premiums for the lowest premium catastrophic plan and the lowest premium subsidized bronze plan, with and without expand premium subsidies for a 45-year-old marketplace enrollee with an income from 250% to 500% FPL. The sample consisted of 1420 counties in 21 healthcare.gov states where catastrophic plans are available in 2025. We weighted premiums by 2025 enrollment above 250% of the FPL ($39 125 dollars in annual income for a single person and $80 375 dollars for a family of four). We identified the lowest premium catastrophic and subsidized bronze plans for each county using the 2025 Qualified Health Plan Landscape File from healthcare.gov. We calculated subsidies for each age and FPL unit using: (1) marketplace age rating multipliers from the CMS; (2) 2025 federal poverty guidelines from the Department of Health and Human Services; (3) the latest marketplace expected contribution percentages from the CMS—2025 for expanded premium tax credits and 2026 for non-expanded premium tax credits; and (4) benchmark plan premiums from the 2025 Qualified Health Plan Landscape File. We calculated the difference between the lowest premium catastrophic plan and the lowest premium subsidized bronze plan. We report differences and 95% confidence intervals at 25-percentage point intervals in Appendix Table S1. Results for enrollees of different ages are shown in Appendix Figure S1.

Expanding catastrophic coverage eligibility will not improve premium affordability for enrollees with incomes from 250% to 400% FPL (Figure 1), with highly limited exceptions. Without ePTCs, a 45-year-old enrollee with an income of 250% FPL will pay $436 per month less on average (95% CI = $419 to $453) for subsidized bronze coverage than catastrophic coverage; at 400% FPL, they will pay $207 less (95% CI = $190 to $224). With ePTCs, they will pay $503 less (95% CI = $486 to $520) at 250% FPL to $280 less (95% CI = $263 to $297) at 400% FPL. Near 400% FPL, catastrophic coverage is cheaper in 0.5% (11) of sample counties with ePTCs and 3.5% (75) without.

Catastrophic coverage is more affordable than bronze coverage for enrollees with incomes above 400% FPL only if ePTCs are not extended (Figure 1). Without ePTCs, catastrophic coverage would be $104 per month (95% CI = $121 to $87) less. However, if ePTCs are extended, subsidized bronze coverage would be $280 (95% CI = $263 to $297) less than catastrophic coverage at 400% FPL, to $173 (95% CI = $156 to $190) less at 500% FPL.

Results are consistent regardless of enrollee age (Appendix Figure S1).

## Discussion

CMS expanding catastrophic coverage eligibility will, with extremely limited exceptions, not improve premium affordability for marketplace enrollees with incomes from 250% to 400% FPL. In the two thirds of counties where catastrophic plans were available in 2025, expanding catastrophic coverage eligibility will improve premium affordability for enrollees above 400% FPL only if ePTCs expire.

Extending ePTCs would improve coverage affordability by hundreds of dollars per month more than expanding catastrophic coverage eligibility. Additionally, extending ePTCs would not require enrollees switch to coverage that may place them at greater vulnerability to catastrophic medical expenses to avoid premium increases.^6,7^ However, extending ePTCs would cost the federal government $350 billion over a decade.^8^

A limitation of our analysis is that 2026 plan premium data are not yet available. Our findings may overestimate the affordability of catastrophic coverage if their premiums rise in response to newly eligible, relatively older enrollees shifting to catastrophic plans.^9^ Future research should examine how policy changes affect 2026 marketplace enrollment and affordability, and how enrollees switching to less robust coverage affect healthcare use and health outcomes.

## Supplementary Material

qxaf202_Supplementary_Data
