# Supplementary material for: Will expanding catastrophic coverage eligibility increase marketplace premium affordability in 2026?
Source: Health Aff Sch. 2025 Oct 23;3(11):qxaf202. doi: 10.1093/haschl/qxaf202 (PMC12596389; doi:10.1093/haschl/qxaf202)
Supplement: qxaf202_Supplementary_Data [file qxaf202_supplementary_data.zip › SUPPLEMENTAL APPENDIX Data and Measures.docx]

**SUPPLEMENTAL APPENDIX** Data and Measures

**Data**

We used the following data sources:

1. Qualified Health Plan Landscape File (2025): Lists premiums and metal levels of all plans offered on healthcare.gov by county. Made available by the Center for Consumer Information and Oversight at <https://data.healthcare.gov/qhp-landscape-files>.
2. Marketplace age rating: Lists age rating factors that are applied to premium based on enrollees’ ages. Published by the Center for Consumer Information and Oversight at <https://www.cms.gov/cciio/programs-and-initiatives/health-insurance-market-reforms/state-rating>.
3. Federal poverty level (2025): Identifies annual incomes by family size at the poverty threshold. Published by the Department of Health and Human Services at [https://www.federalregister.gov/documents/2025/01/17/2025-01377/annual-update-of-the-hhs-poverty-guidelines](https://www.federalregister.gov/documents/2025/01/17/2025-01377/annual-update-of-the-hhs-poverty-guidelines?utm_source=chatgpt.com)
4. Marketplace advanced premium tax credit subsidy expected contribution percentages (2025 and 2026): Establish the percentage of modified adjusted gross income that Marketplace enrollees must pay in excess of advanced premium tax credit subsidies to purchase the benchmark Marketplace plan in their county. Published by the Internal Revenue Service for 2025 at <https://www.irs.gov/pub/irs-drop/rp-24-35.pdf>, and for 2026 at <https://www.irs.gov/pub/irs-drop/rp-25-25.pdf>.
5. Open Enrollment Period Public Use File (2025): Lists Marketplace enrollment by county and FPL level. Published by the Centers for Medicare and Medicaid Services at <https://www.cms.gov/data-research/statistics-trends-reports/marketplace-products/2025-marketplace-open-enrollment-period-public-use-files>

**Measures**

Our analysis used three outcomes, each at the county level. We constructed them as follows:

1. Lowest premium catastrophic plan: We identified the lowest premium catastrophic plan in each county using the Qualified Health Plan Landscape File. We applied the age rating curve to obtain premiums at different ages.
2. Lowest premium bronze plan with premium subsidies: We analogously followed the steps for creating the lowest premium catastrophic plan described above. We then calculated the premium subsidy available to each marketplace enrollee, by age and income, using the federal poverty level data, expected contribution percentage data, and the benchmark plan’s premium as listed in the Qualified Health Plan Landscape File. The benchmark premium is the second lowest premium silver plan. Premium subsidies are equal to the difference between the age-adjusted benchmark plan premium and enrollee’s expected contributions, which are in turn equal to their modified adjusted gross income times their expected contribution percentage. For this measure, we used 2026 expected contribution percentages that do not include expanded premium tax credits.
3. Lowest premium bronze plan with expanded premium subsidies: We repeat all steps as in the prior measure except that we used 2025 expected contribution percentages that do include expanded premium tax credits.

We take weighted means of these measures across sample counties, weighting by marketplace enrollment above 250% FPL using the Open Enrollment Period Public Use File.

**APPENDIX FIGURE A1** Differences between 2025 Mean Monthly Premiums for the Lowest Premium Catastrophic Plan and the Lowest Premium Subsidized Bronze Plan, with and without Expand Premium Subsidies for a 35-, 45-, 55-, and 64-Year-Old Marketplace Enrollee with an Income from 250-500% FPL


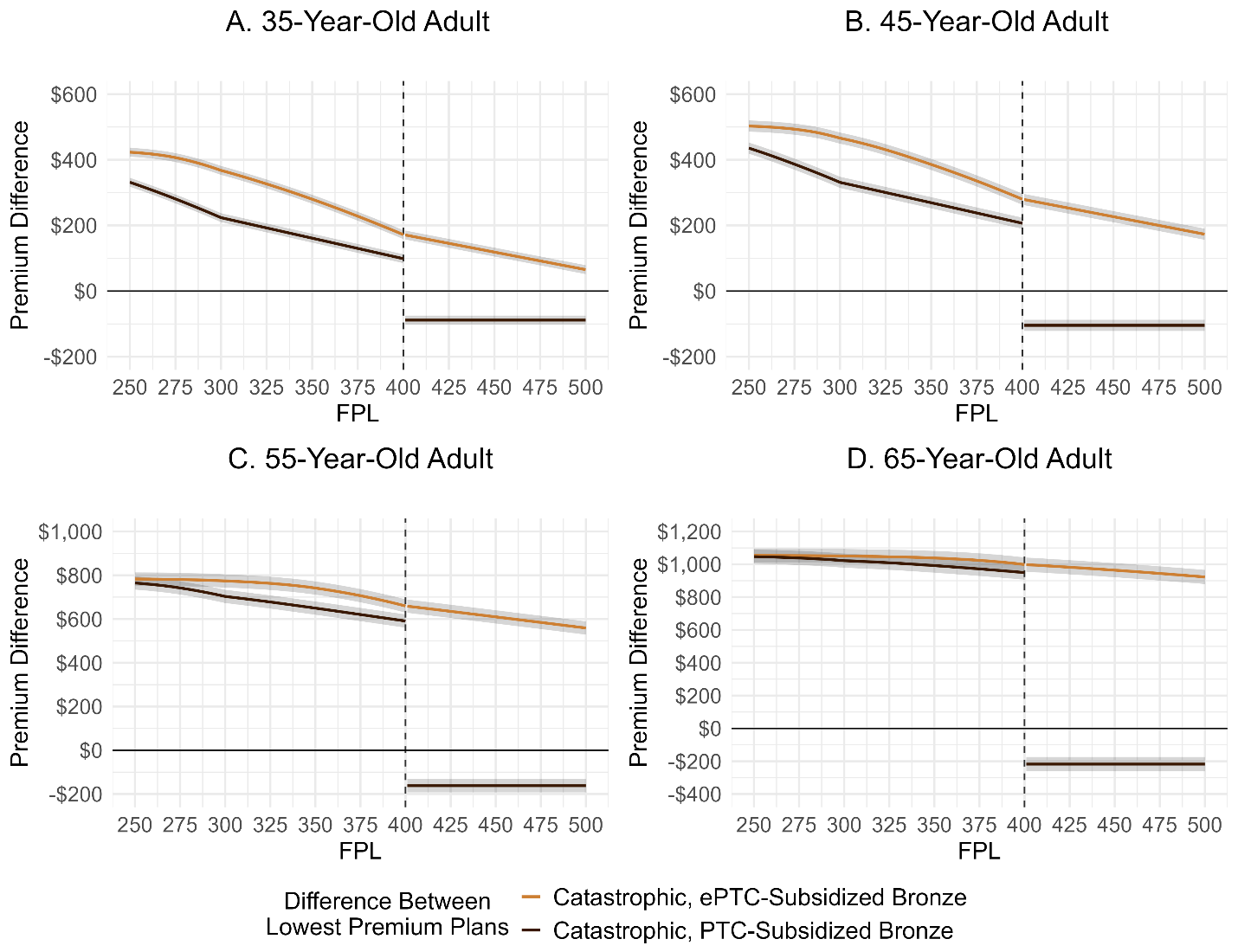


Notes: Panel B is shown in the manuscript. Other panels are analogous to Panel B but for different ages. Note variations in y axes resulting from different premium levels.

**APPENDIX TABLE A1** Differences between 2025 Mean Monthly Premiums for the Lowest Premium Catastrophic Plan and the Lowest Premium Subsidized Bronze Plan, with and without Expand Premium Subsidies for a 45-Year-Old Marketplace Enrollee with an Income from 250-500% FPL, at 25-Percentage Point Intervals

| Income (% FPL) | Mean Monthly Premium Difference, Lowest Premium Catastrophic Plan and Subsidized Bronze Plan (95% CI) | |
| --- | --- | --- |
|  | With ePTC | Without ePTC |
| 250 | $503 ($486, $520) | $436 ($419, $453) |
| 275 | $493 ($476, $510) | $387 ($370, $404) |
| 300 | $466 ($449, $483) | $331 ($314, $348) |
| 325 | $431 ($414, $448) | $300 ($283, $317) |
| 350 | $386 ($369, $403) | $269 ($252, $286) |
| 375 | $336 ($319, $353) | $238 ($221, $255) |
| 400 | $280 ($263, $297) | $207 ($190, $224) |
| 425 | $253 ($236, $270) | -$104 (-$121, -$87) |
| 450 | $227 ($210, $244) | -$104 (-$121, -$87) |
| 475 | $200 ($183, $217) | -$104 (-$121, -$87) |
| 500 | $173 ($156, $190) | -$104 (-$121, -$87) |

Notes. This is a tabular representation of Figure 1.
